# 

**Published:** 1861-01

**Authors:** 


### CONTENTS.

### ORIGINAL COMMUNICATIONS.

PAGE

ARTICLE I.—Doings of Parisian Societies—gathered from the French Journals by one of our Correspondents,........................253

ARTICLE II.—Report of a Case of Pneumonia, to Atlanta Medical Society,........................................................263

ARTICLE III.—Report of a Case of Mania. By F. M. Hunt, M. D., of Liberty Hill, Ga.,..............................................265

### SELECTIONS.

Contributions to Urology,...............................................269

Aphorisms Respecting the Rodent Ulcers,..........................275

Certain Diseased Conditions of the Ovum and its Envelopes, induced by Constitutional Vice in the Mother, as a cause of Abortion,.....278

On Opening the Joints,...................................................286

The Dengue or Break-Bone,..............................................291

Clinical Lectures,...........................................................293

### EDITORIAL AND MISCELLANEOUS.

The New Title and Secession,............................................303

Port Wine,.....................................................................307

Secession,.....................................................................311

Wounds of the Scalp united by the Hair,..............................313

A Handbook of Hospital Practice,.......................................314

Baltimore Journal of Medicine—A New Journal,......................315

Register of Meteorological Observations,..............................316

